# Changes in the Content of Some Groups of Phenolic Compounds and Biological Activity of Extracts of Various Parts of Heather (*Calluna vulgaris* (L.) Hull) at Different Growth Stages

**DOI:** 10.3390/plants9080926

**Published:** 2020-07-22

**Authors:** Victoria Chepel, Valery Lisun, Liubov Skrypnik

**Affiliations:** 1 Laboratory of Natural Antioxidants, Institute of Living Systems, Immanuel Kant Baltic Federal University, Universitetskaya str., 2, 236040 Kaliningrad, Russia; VCHepel@stud.kantiana.ru; 2 Laboratory of Microbiology and Biotechnologies, Institute of Living Systems, Immanuel Kant Baltic Federal University, Universitetskaya str., 2, 236040 Kaliningrad, Russia; VLisun@stud.kantiana.ru

**Keywords:** heather, vegetation, plant organ, rhizome, flowers, flavonoids, hydroxycinnamic acids, anthocyanins, antioxidant activity, antibacterial activity

## Abstract

Heather (*Calluna vulgaris* (L.) Hull.) is noted for a diverse chemical composition and a broad range of biological activity. The current study was aimed at monitoring changes in the accumulation of certain groups of phenolic compounds in various organs of heather (leaves, stems, roots, rhizomes, flowers, and seeds) at different growth stages (vegetative, floral budding, flowering, and seed ripening) as well as studying antioxidant (employing the DPPH and FRAP assays) and antibacterial activity of its extracts. The highest total amount of phenolic compounds, tannins, flavonoids, hydroxycinnamic acids, and proanthocyanidins was detected in leaves and roots at all growth stages, except for the flowering stage. At the flowering stage, the highest content of some groups of phenolic compounds (flavonoids, proanthocyanidins, and anthocyanins) was observed in flowers. Highest antioxidant activity was recorded for the flower extracts (about 500 mg of ascorbic acid equivalents per gram according to the DPPH assay) and for the leaf extract at the ripening stage (about 350 mg of ascorbic acid equivalents per gram according to the FRAP assay). Strong correlation was noted between antioxidant activity (DPPH) and the content of anthocyanins (*r* = 0.75, *p* ≤ 0.01) as well as between antioxidant activity (FRAP) and the total content of phenolic compounds (*r* = 0.77, *p* ≤ 0.01). Leaf extracts and stem extracts turned out to perform antibacterial action against both gram-negative and gram-positive bacteria, whereas root extracts appeared to be active only against *B. subtilis*, and rhizome extracts against *E. coli*.

## 1. Introduction

Heather (*Calluna vulgaris* (L.) Hull.) belongs to the family of vacciniaceous plants (Ericaceae Juss.) and the monotypic genus *Calluna* Salisb. [1]. Heather is a relict evergreen dwarf shrub and widely distributed in Europe. The range of its growing area also comprises the British Isles, Iceland, North Africa, North America, Australia, and New Zealand [2,3].

Heather, as an oligotrophic plant, grows in soils poor in minerals, and is representative of the marganophilic plants [4,5]. The tolerance of heather to temperature, altitudinal zonation, as well as the length of the growing season varies widely in range [3].

Heather has long been used as a medicinal herb active against rheumatism, arthritis, as well as an antiseptic, choleretic, vulnerary, and expectorant [6,7]. Moreover, its decoction is applicable for treating the diseases of the urinary tract, which is indicative of its anti-inflammatory, diuretic, and antimicrobial properties [8,9]. In addition, it is used as a preventive and therapeutic agent in increased irritability, anxiety, sleep disturbances, and decreased performance, all of which may be owing to its inhibitory effects on monoamine oxidase-A (MAO-A) [10,11]. The carried out pharmacological studies of extracts of the aerial parts of the plant revealed its antioxidant, antiviral, hepatoprotective, cardioprotective, cytotoxic, genoprotective, and antiproliferative effects [12,13,14,15,16].

The wide variety of medicinal properties of heather is determined by the biologically active substances present, especially the phenolic compounds [17].

The phenolic compounds found in heather include monomeric phenols (hydroquinone and arbutin of up to 1.5%); phenolcarboxylic, hydroxycinnamic, and hydroxybenzoic acids; coumarins, and tocopherol (vitamin E). Among the flavonoids in heather plants are representatives of subclasses, such as flavonols, dihydroflavonols, flavones and isoflavones, flavonones, chromones, catechins, tannins, proanthocyanidins, and anthocyanins [18]. The most distinct flavonoids of heather are quercetin and its glycosides alongside callunin and kempferol-3-β-D-galactoside [19]. It should be noted that, in addition to phenolic compounds in the leaves and flowers of heather, terpenoids (lupeol, ursolic, and oleanolic acids) have been identified and determine the antitumor effect of heather extracts, as well as organic acids (ascorbic, oxalic), β-carotene (provitamin A), and steroids [17,20].

Biosynthesis and accumulation of secondary metabolites in plants, including phenolic compounds, depend on a number of factors, such as species exclusiveness, vegetation period, and growing conditions (climatic factors, altitude, and soil properties) [21,22,23,24]. Despite the fact that the phenolic compounds get synthesized in all parts of the plant, their content in various organs change in the course of the plant growth and development. This is primarily linked to an increase or decrease in the expression of the genes encoding the activity of enzymes involved in the biosynthesis of phenolic compounds [25]. Thus, taking *Dryopteris erythrosora* as an example, it was proved that the total content of flavonoids in the stems is higher than that in the leaves, and reaches its maximum in June [26]. In *Chelidonium majus*, the highest concentration of flavonoids was revealed in the leaves before flowering whilst the lowest during flowering [27]. With regard to *Vaccinium ashei*, also of the Ericaceae family and a closely related species to heather, it was found that its leaves collected in March possessed the highest content of phenolic compounds and flavonoids [28].

Even though the phytochemical composition of heather is quite well researched [12,16,17,18,19,29,30], studies on the change in content of the phenolic compounds in various plant organs (leaves, stems, roots, rhizomes, flowers, and seeds) at various stages of ontogenesis did not take place previously. The authors of the studies available to date researched either the seasonal variation in phenolic compound content in a whole aerial part of the plant or in various organs of the plant at one vegetation phase (mainly at the flowering stage). A study on the biosynthesis and accumulation of different groups of phenolic compounds in various parts of *C. vulgaris* can provide valuable evidence to promote the best utilization of this plant.

In this regard, the purpose of this study was to research the dynamic accumulation of some groups of phenolic compounds in the organs of common heather (*C. vulgaris*) at different growth stages, as well as the analysis of the antioxidant and antibacterial activity of its extracts against *Escherichia coli* and *Bacillus subtilis.*

## 2. Results

### 2.1. Content of Phenolic Compounds

#### 2.1.1. Total Phenolic Compounds Content

**Figure 1:**
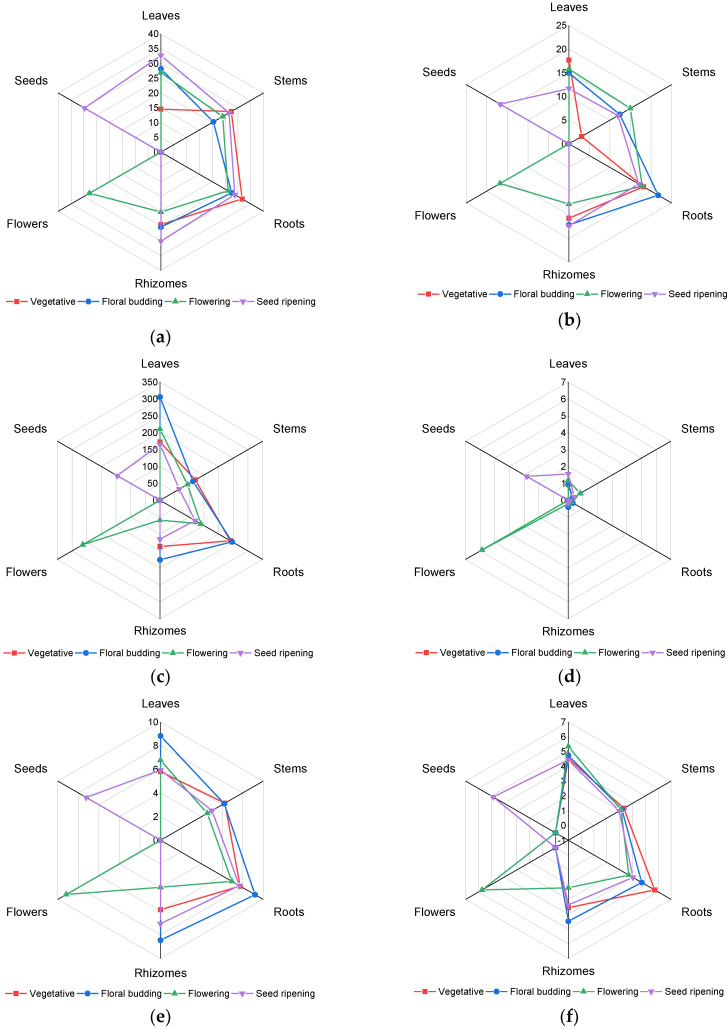
Changes in content of phenolic compounds in various parts of heather collected at different growth stages: (**a**) total phenolic compounds; (**b**) tannins; (**c**) flavonoids; (**d**) anthocyanins; (**e**) proanthocyanidins; (**f**) hydroxycinnamic acids. The content of all the studied compounds are given in the plots in mg g^−1^ DW.

Heather is defined by a high content of phenolic compounds, which, along with triterpenes, are the core components in this plant responsible for its diverse biological activity and pharmacological value. As our studies revealed, the distribution of phenolic compounds in heather organs were specific to the growth stage (Figure 1a; Table 1). Thus, for example, at the vegetative stage, the roots stood out for their maximum content thereof, whilst the leaves contained the minimum value. As the plant developed, redistribution of the phenolic compounds took place. At the stage of floral budding, the highest amount of phenolic compounds was detected in leaves and roots. At the stage of flowering, the highest content of phenolics was observed in the flowers, leaves, and roots. The highest content of phenolic compounds at the stage of seed ripening was established in the leaves. Besides, it should be noted that at the stage of seed ripening, overall, an increase was observed in the value of the total content of the phenolic compounds in all plant organs, including the fruit formed in the flowers. This increase was especially prominent in the rhizomes: along with moving from the stage of flowering into the stage of seed ripening, the content increased by 30%.

#### 2.1.2. Total Tannins Content

The results of the research on accumulation of tannins in different parts of heather versus the growth stage is shown in Figure 1b. Overall, the roots and rhizomes, as well as the flowers and seeds, maintain approximately equal high values of tannins throughout the vegetative period of heather. The lowest content of tannins at the vegetative stage was detected in the stems, which, in the course of the plant development, increased by about four times. As for the leaves, there was a decrease in the level of tannins observed at the stage of seed ripening.

#### 2.1.3. Total Flavonoids Content

The analysis of the dynamics of the total flavonoid content revealed that at the growth stage the roots contained the maximum flavonoid content whilst the stems featured the minimum content (Figure 1c). In the leaves there was an increase in content of the flavonoids from the vegetative stage to the floral budding stage followed by a gradual decrease in content. It was in the leaves at the floral budding stage that the maximum flavonoid content was detected amongst all the studied samples. At the flowering stage, a decrease in flavonoid content by about a third was observed in all studied organs. Moreover, the maximum content of the flavonoids was revealed in the flowers. The stage of seed ripening was defined by a relatively low content of flavonoids in all the studied organs if compared to the other phases of plant growth.

#### 2.1.4. Total Anthocyanins Content

Accumulation of the anthocyanin pigments in various parts of heather is exhibited in Figure 1d. The maximum value of anthocyanin pigments was spotted in the flowers: 5.88 ± 0.13 mg g^−1^. The high content of anthocyanins in the flowers determines their rich coloring to attract pollinating insects, and that is especially important in view of plant propagation. High levels of anthocyanins are also found in the seeds. Still, it was about two times lower than in the flowers. The leaves revealed the average content of anthocyanin pigments, which increased in the course of the plant development. In the subterraneous organs of heather, namely the roots and rhizomes, the content of the anthocyanins was at its lowest.

#### 2.1.5. Proanthocyanidins Content

Accumulation of the proanthocyanidins in the heather organs was predetermined by its vegetation phase (Figure 1e). For instance, the roots at the vegetative and floral budding stages were defined by a maximum content of proanthocyanidins. At the same time, at the floral budding stage, a large amount of proanthocyanidins was also spotted in the leaves and rhizomes. At the flowering stage, the level of proanthocyanidins decreased in all parts of the plant by approximately 15–20% (by 52% in the rhizomes), while the flowers formed at this stage possessed the highest content of proanthocyanidins. At the stage of seed ripening, the level of proanthocyanidins in the seeds stood roughly at the same level as that of the subterranean organs (7.06–7.67 mg g^−1^).

#### 2.1.6. Total Hydroxycinnamic Acids Content

The outcome of the research on accumulation of hydroxycinnamic acids in various organs of heather is shown in Figure 1f. The analysis revealed that accumulation of hydroxycinnamic acids in the heather organs depended on the vegetation phase. In the stems, with the change in vegetation phases, there were no significant differences in view of the content of hydroxycinnamic acids. In the leaves there was a gradual increase in hydroxycinnamic acids observed from the growth stage to the flowering stage, and its decrease at the stage of seed ripening. However, for instance, in the roots and rhizomes, a decrease in the content of hydroxycinnamic acids from the stage of floral budding to flowering was witnessed; still, throughout the period of seed ripening the level of hydroxycinnamic acids increased, particularly by approximately 50% in the rhizomes. Thus, in the subterranean organs, the highest content of hydroxycinnamic acids was observed at the vegetative and floral budding stages, whilst in the areal parts of the plant it was a characteristic feature of the flowering stage.

### 2.2. Principal Component Analysis (PCA) of Phenolic Compounds Content

**Figure 2:**
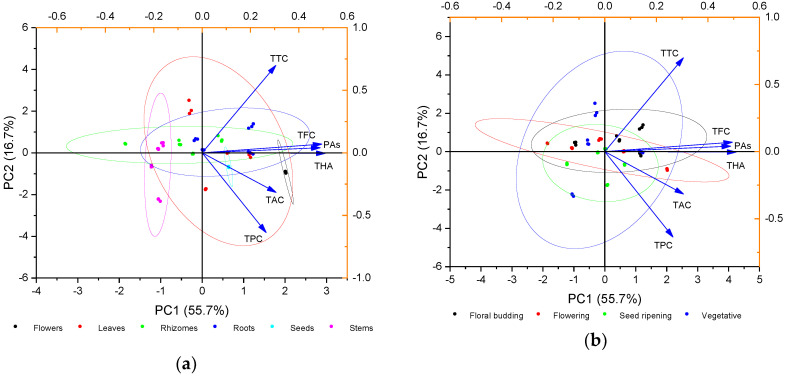
Principal component analysis (PCA) biplot of the phenolic compounds content grouped by (**a**) plant part and (**b**) growth stage. Color ellipses indicate the 95% confidence interval for each group.

The obtained data on the content of the phenolic compounds in various parts of heather versus its phase of vegetation were statistically processed employing the PCA method. The outcome of the analysis as a biplot is given in Figure 2. The total six principle components can explain 100% of the total variance. The first principal component (PC1) represented 55.7% of the variance, and the second principal component (PC2) represented 16.7 % of the variance. The next two principal components represented 14.1% (PC3) and 7.9% (PC4) of the variance, respectively. The 95% bootstrapped confidence interval for PC1 was 44.3–68.2 % (N = 999). To further identify the contributors to the principle components, the factor loadings of PC1 and PC2 were compared. In PC1, the corresponding loading was positive for all phenolic compounds. Flavonoids, proanthocyanidins, and hydroxycinnamic acids revealed the closest bond regarding their accumulation in heather plants.

For the needs of presenting the outcome by means of the biplot, the data were grouped by the plant part (Figure 2a) and its vegetation phase (Figure 2b). The greatest variability in content of the phenolic compounds was revealed in the leaves. As for the subterranean organs (roots and rhizomes), the patterns of accumulation of the phenolic compounds were similar. Grouping by vegetation stage proved that the utmost scatter of data was characteristic of the vegetative stage. The closest bond in accumulation of the phenolic compounds was witnessed at the stages of flowering and seed ripening.

### 2.3. Antioxidant Activity

**Figure 3:**
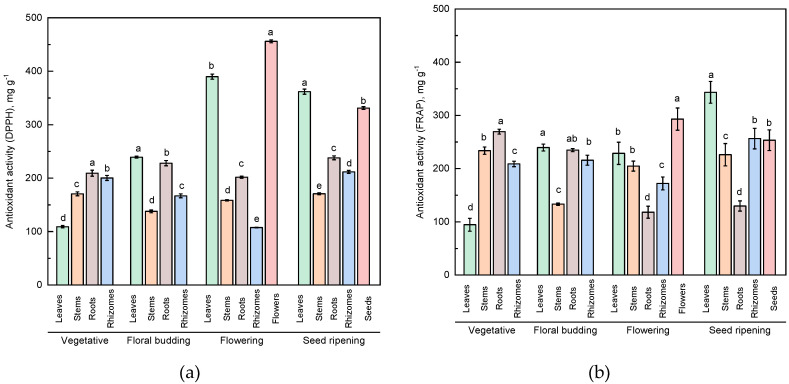
Antioxidant activity of the extracts from different parts of heather collected at four growth stages. (**a**) Antioxidant activity according to the DPPH assay and (**b**) antioxidant activity according to the FRAP assay. Data were evaluated via one-way ANOVA separately for each growth stage followed by a Tukey test. Different letters indicate significant difference among different heather parts at *p* ≤ 0.05.

The antioxidant activity of plant extracts depends on the presence of various compounds with a different mechanism of antioxidant action. In this regard, in this study, two methods to evaluate the antioxidant activity of heather extracts were employed. The DPPH assay is based on the ability of antioxidants to bind radicals. The FRAP assay serves as an indicator of the reducing power of the plant extracts. To compare the data obtained by these two assays, ascorbic acid as a standard was used. Despite the fact that the absolute values of antioxidant activity measured by the DPPH and FRAP assays were different, the changes in antioxidant activity of the heather extracts were strongly preconditioned by both the plant organ and its phase of collection (Figure 3a,b). Thus, the antioxidant activity of the leaf extracts at the vegetative stage according to the DPPH and FRAP assays was at its lowest if compared to the other organs. Still, at the flowering and seed ripening stages, it increased by about four times. A relatively high antioxidant activity at the vegetative stage was characteristic of the extracts of roots and rhizomes (according to the DPPH assay) and stems and roots (according to the FRAP assay). Moreover, with respect to the roots, there was no significant variation in antioxidant activity measured by the DPPH assay versus the growth stage, while for the rhizomes a decrease in antioxidant activity from the vegetative stage to the flowering stage was observed with its further increase at the stage of seed ripening. The stem extracts revealed medium to low levels of antioxidant activity according the DPPH test at all phases of heather collection. The antioxidant activity of the stem extracts measured using the FRAP assay was at the medium level at all growth stages. It should be noted that the extracts of the generative parts of heather demonstrated high antioxidant activity. The antioxidant activity at its maximum was detected for the flower extracts of heather (about 500 mg g^−1^ according to the DPPH assay). For the seeds, this parameter stood at 331 mg g^−1^. The flower extracts revealed a high antioxidant activity, measured using the FRAP assay. However, the extracts from seeds were characterized by a medium ferric-reducing power.

Antioxidant activity depends on the content of various classes of phytocomponents in the samples. Normally, the phenolic compounds are responsible for a significant contribution to the antioxidant activity of the extracts. The correlation analysis performed during this study revealed a positive relation between the content of different groups of phenolic compounds and the antioxidant activity of the heather extracts (Table 2). The high degree of correlation was registered between the antioxidant activity measured using the DPPH assay and the content of the anthocyanins (*r* = 0.75, *p* ≤ 0.01). The average degree of correlation between the antioxidant activity (DPPH assay) and the content of the hydroxycinnamic acids (*r* = 0.68, *p* ≤ 0.01), as well as the total amount of phenolic compounds (*r* = 0.65, *p* ≤ 0.01) and flavonoids (*r* = 0.52, *p* ≤ 0.01) was established. For antioxidant activity measured using the FRAP assay, the highest degree of correlation was established for total phenolic content (*r* = 0.77, *p* ≤ 0.01). The coefficient of correlation between the antioxidant activity values obtained using different assays was statistically significant (*r* = 0.63, *p* ≤ 0.01).

### 2.4. Antibacterial Activity

Antibacterial activity of the extracts from various parts of heather collected at its various growth phases growth was studied in relation to a representative of the Gram-negative bacteria, *Escherichia coli*, and in relation to a representative of the Gram-positive bacteria, *Bacillus subtilis*. The results of the study are exhibited in Table 3. Given the results presented in the table, it is apparent that the leaf extracts are effective against *E. coli* (at the vegetative, floral budding, and seed ripening stages) and inhibit the growth of *B. subtilis* only at the stage of floral budding.

Extracts from the stems at the vegetative and floral budding stages also proved to be active against both Gram-negative and Gram-positive bacteria. At that, the extract from stems at the vegetative stage proved to have quite a pronounced antibacterial effect against *E. coli* (the inhibition zone reached 11 mm at a 50 mg m^−1^ concentration of extract) and against *B. subtilis* at the floral budding stage (the inhibition zone reached 10 mm at a 50 mg mL^−1^ concentration of the extract).

Extracts from the roots at the vegetative stage did not show any antibacterial activity. At the stages of floral budding and flowering, the extracts from the root revealed a weak antibacterial effect against *B. subtilis* (with the inhibition zone of 9 to 9.5 mm at 50 mg mL^−1^ concentration). At the same time, among all the extracts, the ones from the heather roots at the stage of seed ripening revealed the greatest antibacterial effect against *E. coli* (with the inhibition zone at a 50 mg/mL concentration reaching 12 mm) and quite a pronounced activity against *B. subtilis* (the inhibition zone reached 10 mm at a 50 mg mL^−1^ concentration).

Extracts from the rhizomes revealed some antibacterial effect by showing a weak activity against *B. subtilis* only at the stage of floral budding.

The generative parts of the plant (its flowers and seeds) manifested a weak antibacterial effect and only against the Gram-negative bacterium *E. coli*. Still, extracts from the flowers proved to possess the weakest inhibitory effect (with an inhibition zone of 7.5 mm at a 50 mg mL^−1^ concentration of the extract).

In general, it should be mentioned that the floral budding stage is the only stage at which the antibacterial activity of the extracts from all the studied parts of the plant is detected. In addition, the leaves and stems proved to have an antibacterial effect against both Gram-negative and Gram-positive bacteria, whilst for the roots it only applies to *B. subtilis* and for the rhizomes to *E. coli*.

## 3. Discussion

Heather stands out for its rich and varied chemical composition, and features a wide range of biological activity. Nonetheless, the accumulation of biologically active substances in heather plants depends heavily on a number of factors related to the plant itself (the vegetation phase and the organ) as well as on its exposure to diverse environmental conditions (climatic factors, altitude, and soil properties) [21,22,23,24]. Our studies proved that the accumulation of certain classes of phenolic compounds in various organs of heather was determined by the phase of its vegetation. However, the two-way ANOVA analysis revealed a consistent pattern of the utmost accumulation of some groups of phenolic compounds in the leaves and roots at all phases of vegetation growth (Table A1). As discussed, the biosynthesis and accumulation of flavonoids proceed in each organ in its own way. Nonetheless, the confidence of these substances to be in the actively metabolizing organs was established, namely in the leaves that provide the entire plant with the secondary products of photosynthesis through the phloem [31].

The roots, featuring a high content of the phenolic compounds, also perform a number of functions that determine accumulation of these secondary metabolites in them. The plant roots use polyphenols to adapt to the rhizosphere, wherein the arbuscular mycorrhizal fungi reside and with which heather forms an ericoid mycorrhiza [32]. In particular, the flavonoids have a positive effect on symbiosis by stimulating spore germination, hyphae development, as well as root colonization. The mycorrhiza increases the influx of assimilates to the roots [33]. An additional role of phenolic compounds in heather roots is associated with the mineral nutrition and absorption of iron from the soil. In the bogs where heather grows, there are iron deposits in the form of oxyferry complexes, which are insoluble and therefore inaccessible to absorption by plants. Plants adapt by releasing hydroxycinnamic acids and catechins into the rhizosphere by their roots helping to restore Fe(III) to Fe(II) and making it available for absorption by the roots [33]. The results of our analysis confirms the increased accumulation of phenolic compounds by the roots at various vegetation phases. This assumption is likely to be supported by the reasons given above.

To date there is no clarity on the issue of whether phenolic compounds can travel in a plant. Previously, it was assumed that the flavonoids synthesized in individual parts of the plant are not transported, whilst only the initial fragments can travel to ensure biosynthesis of the flavonoid structure [34]. However, a recent study has proved that the outflow of flavonoids from the stems to the leaves is possible, so too from the leaves into the phloem and further movement around it, as well as from the roots to other parts of the plant [35]. Other authors came forth with the unidirectional movement and tissue specificity for the accumulation of flavonoids. There are also beliefs that the distribution of flavonoids is intermediated by an active process instead of passive diffusion, and possibly under the influence of a carrier protein [36]. The probability of phenols moving from the leaves into phloem, from it further into the xylem, and then from there with an upward current to the top of the plant, is associated with fruit formation [37]. The study of *Arabidopsis thaliana* revealed that flavonoid products accumulate inside the cells and are not present in areas between the cells. This fact suggests that the movement of these molecules over large distances occurs through symplasts, and so they can be transported on a selective basis throughout the plant from one of its organs to another [38]. Flavonoids are hardly produced in plants or organs grown in the dark due to the condition that the expression of the gene is highly dependent on the light [38]. However, the synthesis and further accumulation of polyphenols in the roots is confirmed by a correlation between the clusters of specific transcripts of genes or enzymes and metabolites of biosynthesis [39].

Change in biosynthesis and accumulation of some groups of phenolic compounds in the course of heather growth and development can be supported by the growth differentiation balance hypothesis and the carbon nutrient balance hypothesis. Growth processes dominate the differentiation or production of secondary metabolites and the phenolic compounds in particular, since the product of photosynthesis, i.e., amino acid phenylalanine, is a common precursor for both protein synthesis and phenolic compounds. If the plant growth is active, and therefore requiring a large amount of carbon for the synthesis of amino acids, then the synthesis of secondary metabolites is reduced [40]. Overall, in our study, based on the outcome of the two-way analysis of variance, the content of the various groups of phenolic compounds, except for hydroxycinnamic acids, was at a relatively low level at the vegetative stage. The content of hydroxycinnamic acids was at its highest at the vegetative stage, when especially active plant growth was recorded. However, this class of compound, when compared to the other studied groups of phenolic compounds, is represented by the simplest compounds in view of their structure, and their biosynthesis requires consumption of less energy. A decrease in level of hydroxycinnamic acid over the period of plant development up to the stages of flowering and seed ripening may be representative of the fact that monomeric phenols serve as the initial components for lignin synthesis, with the latter being the main polymer of the plant-supporting tissues and an important component of the secondary cell wall [41].

An increase in the content of flavonoids during the transition of the plant to its generative development, their maximum accumulation before flowering at the floral budding stage, and their decreasing content at the flowering stage indicate an expense of these compounds on the physiological and adaptive processes [42]. A decrease in the content of flavonoids at the seed ripening stage can be associated not only with the slowdown in biosynthesis, but rather with the activation of the oxidation enzymes of the flavonoids. It is believed that the process of oxidation of the enzymes is most active in leaves, fruit, and stems [43]. The data obtained in our study support that hypothesis. The level of flavonoids after flowering and at the seed ripening stage remained unchanged in the roots, while it was rapidly decreasing in the leaves and reproductive organs.

The antioxidant capacity of the phenolic compounds is determined primarily by their aromatic structure with a system of conjugated double bonds, as well as by the presence of a hydroxyl group in their structure. Owing to these structural features, detachment of hydrogen and neutralization of free radicals and other reactive oxygen intermediates becomes achievable [44,45]. The results obtained in this study on the relationship in the accumulation of the total phenolics and antioxidant activity of heather extracts are consistent with previously obtained data presented in the work by Dróżdż et al. [30]. According to the DPPH assay revealed in our study, the strongest correlation was between anthocyanins and antioxidant activity. The obtained result is primarily associated with a high level of anthocyanins in heather flowers and a high antioxidant activity of the extracts from them. It is known, per se, that anthocyanins make a significant contribution to the antioxidant activity of berries [46], as well as other colored plant products [47].

The study discussed in this paper proved that, in overall, heather extracts were more active against *E. coli* than against *B. subtilis*. Thus, antibacterial activity against *E. coli* was performed by leaf, stem, flower and seed extracts at different growth stages as well as by root extracts at the seed ripening stage. To investigate antibacterial activity in this study, heather extracts were prepared using 60% water solution of methanol. In the current study, aqueous ethanol solution was used for detecting the content of some groups of phenolic compounds and antioxidant activity, while aqueous methanol solution was used for indicating antibacterial activity based on the literature [48,49] proving that both solutions appear to be good solvents for phenolic compounds, alkaloids, and saponins that are able to perform antibacterial activity. However, the available published data on the effect of phenolic compounds on gram-negative bacteria turn out to be rather contradictory. Even though some authors previously claimed that phenolic compounds do not significantly affect Gram-negative bacteria due to the structure of the cell wall of the latter, other studies examined three action mechanisms of flavonoids affecting Gram-negative bacteria, namely inhibition of nucleic acid synthesis, damage to the cytoplasmic membrane, and inhibition of energy metabolism. The authors discussed that flavonoids could inhibit the hydrophobic surfaces of bacterial membranes with regard to Gram-negative bacteria, most likely owing to their ability to form complexes with extracellular and soluble proteins, as well as with cell walls, and therefore inhibit the formation of biofilms [50]. Despite the structural similarity of various groups of flavonoids, there is a selective activity in relation to various microorganisms. Some authors confirm that the degree of hydroxylation can affect the antimicrobial activity of such phenolic compounds, which indicates that, the more polar flavonoids exist in the extract, the greater the antibacterial effect thereof. For instance, an antibacterial effect was shown for rutin by inhibiting biofilm formation against both Gram-positive (*S. aureus*) and Gram-negative bacteria (*E. coli*). For the other flavonoid compounds of the heather composition, it was shown, to the same extent, that quercetin was active against *B. subtilis*; in turn, myricetrin was the most active compound against *E. coli* [51]. So, the available data allow us to conclude that various groups of flavonoids in heather extracts could be active against the Gram-negative and Gram-positive bacteria tested in the present study.

Overall, it should be noted that the antibacterial activity of all the tested heather extracts was generally weak. However, for a more accurate interpretation of the data on antibacterial activity obtained in this work, additional studies are required. Since the detailed chemical composition of heather was not discussed in this study, more research is needed to identify, expose, and evaluate the antimicrobial activity of individual heather compounds.

## 4. Materials and Methods

### 4.1. Plant Material

**Figure 4:**
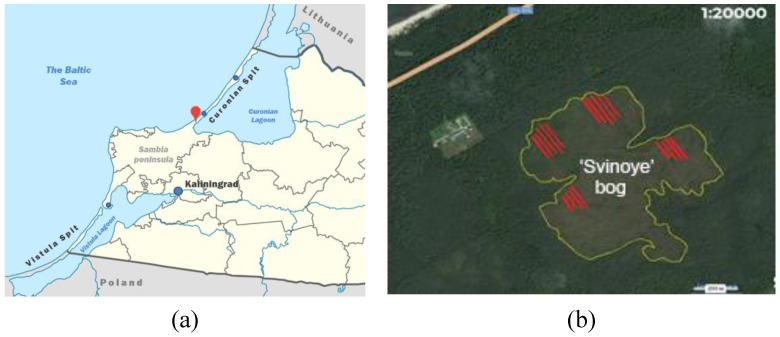
A map showing the places where samples of heather were collected: (**a**) the red point indicate the location of Svinoye bog on Curonian Spit; and (**b**) the trial plots marked with red patterns.

The collection of samples of heather took place from May to October of 2019. The material was gathered from four sample areas on the Svinoye sphagnous raised bog (formerly known as Cranzer Moor) located within the borders of the Curonian Spit National Park (Kaliningrad Region, Russia). The total area of the bog is about 50 ha, and the lot of each sample area was 0.25 ha (Figure 4). From each sample area there were 10 shrubs collected. The collected plants were mixed and represent an averaged sample. The collection of plant material was carried out at four phenological stages: vegetative stage (Stage 39 according to the BBCH scale), floral budding (Stage 55 according to the BBCH scale), full flowering (Stage 65 according to the BBCH scale), and ripening of seeds (Stage 89 according to the BBCH scale). Once collected, the plants were disjointed into separate organs: leaves, stems, roots, and rhizomes (at all stages). At the stage of flowering, the flowers were separated as well and likewise the seeds were dispersed of at the stage of seed ripening. Further, the plant material was dried in air to a constant weight and crushed to the size of particles passing through a sieve with a hole diameter of 1 mm (Pulverisette 7, Fritsch, Germany) afterwards. The dried samples underwent the subsequent analysis to determine the phenolic compounds content and was also used for preparation of the extracts to test the antibacterial and antioxidant activity.

### 4.2. Determination of Phenolic Compounds

#### 4.2.1. Extraction of Phenolic Compounds

The phenolic compounds, except for anthocyanins, were extracted from the crushed dry plant material by a 70% ethanol water solution. A 1 g sample of plant material was placed in a round-bottomed flask with about 20 mL of 70% ethanol added and heated at 60 °C in a water bath under reflux for 1 h. Then the mixture was filtered into a volumetric flask. The procedure of extraction was repeated three times. The obtained extract portions were mixed together and brought to 100 mL by 70% ethanol.

In order to extract the anthocyanins, a 1% hydrochloric acid solution was used. A 0.3 g sample of the dried and crushed plant material was homogenized with 10 mL of 1% HCl. The extraction was performed at room temperature and repeated three times for 60 min in total. The homogeneous mixture was centrifuged for 30 min at 4500 rpm. The supernatant was taken to determine the anthocyanin pigments.

#### 4.2.2. Total Phenolics Content (TPC)

The total content of phenolic compounds was determined spectrophotometrically by means of the Folin–Ciocalteu phenol reagent [52]. The solution was blended by adding 300 μL of the Folin–Ciocalteu phenol reagent, 3 mL of a 7.5% Na_2_CO_3_ solution, and 3 mL of double-distilled water to 100 μL of the extract or 100 μL of a standard solution of gallic acid. Optical absorption of the reaction mixture was measured at a wavelength of 720 nm. The total content of phenolic compounds was calculated by means of a calibration graph plotted against gallic acid, and given in mg of gallic acid equivalents per gram of dry weight.

#### 4.2.3. Total Tannins Content (TTC)

The total tannins content (TTC) was determined by the Prussian Blue method [53] with some modifications, as described previously [54]. Briefly, 0.24 g of casein was added to 10 mL of ethanol extract, and then mixed and incubated at 30 °C for 1 h. Then the solution underwent filtering. The content of the polyphenols was determined in the initial extract as well as in the filtrate employing the Prussian Blue method. In order to do so, 250 μL of the extract or standard solution of gallic acid was mixed with 25 mL of distilled water, and 3 mL of a 0.5 M solution of FeCl_3_ and 3 mL of 0.008 M K_3_Fe(CN)_6_ were added. Optical absorption of the solutions was measured at 720 nm after incubation for 15 min. The content of the tannins was taken as the content of the polyphenols adsorbed on casein. The total tannin content was expressed in mg of gallic acid equivalents per gram of dry weight.

#### 4.2.4. Total Flavonoids Content (TFC)

Determination of the total amount of flavonoids was carried out as reported [55]. The test sample or standard solution of rutin (100 μL) was mixed with 300 μL of 5% NaNO_2_ and incubated for 5 min. Then 300 μL of 10% AlCl_3_ was poured in, and the reaction mixture was incubated for 6 min. Further, 2 mL of 1 M NaOH was added, and the mixture was brought to 10 mL by distilled water. Optical absorption of the solutions was measured at 510 nm. The total flavonoids content was calculated using a calibration curve, and then expressed in mg rutin equivalents per gram of dry weight.

#### 4.2.5. Total Anthocyanins Content (TAC)

The total content of anthocyanins was determined spectrophotometrically. The anthocyanins were extracted by a 1% hydrochloric acid solution (as described above). Optical absorption of the anthocyanin solutions was measured at 510 nm. To correct for the content of green pigments, P.V. Maslennikov suggested to consider the optical density of the extracts obtained at 657 nm. The content of anthocyanins was expressed in mg of cyanidin-3-glucoside equivalents per gram of dry weight [56].

#### 4.2.6. Total Proanthocyanidins Content (PAs)

The proanthocyanidins content (PAs) was determined by a buthanol-hydrochloric acid assay [57]. The reaction mixture consisted of 9 mL of acidified butanol containing iron sulfate (77 mg FeSO_4_ 7H_2_O in 500 mL HCl/BuOH (2/3)) and 1 mL of ethanol extract. The tubes were capped and placed in a water bath to keep at 95 °C for 30 min. Optical absorption of the solutions was measured at 520 nm. The total content of proanthocyanidins was given in mg of cyanidin equivalents per gram of dry weight.

#### 4.2.7. Total Hydroxycinnamic Acids Content (THA)

Determination of the hydroxycinnamic acids content was carried out as reported [58]. The reaction mixture consisted of 1 mL of extract, 2 mL of 0.5 M HCl, 2 mL of reagent obtained by blending nitrite and sodium molybdate (at 1:1), and 2 mL of 8.5% NaOH. The entire volume of the solution was adjusted to 10 mL by distilled water. Optical absorption of the solution was measured at 505 nm. The hydroxycinnamic acids content was calculated by means of a calibration curve, for the plotting of which the standard solutions of chlorogenic acid were employed. The content of hydroxycinnamic acids was given in mg of chlorogenic acid equivalents per gram of dry weight.

### 4.3. Assessment of Antioxidant Activity (AOA)

The antioxidant activity (AOA) of ethanolic extracts of heather obtained as described above was measured by DPPH and FRAP assays. For determination of AOA by DPPH-assay, each extract was mixed with a 2.85 mL freshly prepared 0.1 mM solution of 1,1-diphenyl-2-picrylhydrazyl (DPPH) radical in 96% EtOH. The sample was incubated for 30 min at room temperature in darkness. The reduction of absorbance at 515 nm was measured spectrophotometrically [54]. In the FRAP assay, the reaction was started by mixing 3.0 mL of the FRAP reagent with 100 μL of the investigated extract. The FRAP reagent was freshly prepared by mixing 10 parts of 0.3 M acetate buffer (pH 3.6), 1 part of 10 mM 2,4,6-tripyridyl-triazine (TPTZ) in 40 mM HCl, and 1 part of 20 mM FeCl_3_ × 6H_2_O in dH_2_O. After a 10 min incubation at 37 °C in darkness, the absorbance was measured at 593 nm [59].

As external standard in DPPH- and FRAP-assays were used ascorbic acid solutions in different concentrations. The antioxidant activity was expressed as mg of ascorbic acid equivalents per gram of dry weight.

The absorbance in all spectrophotometric assays was measured by using a UV-3600 spectrophotometer (Shimadzu, Kyoto, Japan).

### 4.4. Assessment of Antibacterial Activity

#### 4.4.1. Plant Extract Preparation

The crushed parts of the plant (1 g) were transferred to a falcon with 10 mL of 70% methanol to extract the active compounds. The plant material was extracted by stirring while placed in a multi-rotator (Multi Bio RS-24, Latvia) for 48 h at room temperature. Then, the homogenized mixture was centrifuged for 60 min. The obtained supernatant was evaporated to dryness using a vacuum concentrator. The obtained dry extract was weighed and dissolved by 10% dimethyl sulfoxide (DMSO) [60].

#### 4.4.2. Bacteria Culture

Two types of bacteria were involved to test the plant extracts, namely, the Gram-positive *Bacillus subtilis* (B-9865) and Gram-negative *Escherichia coli* (DH5α). For planting into a liquid culture, a single colony of the test organism was taken. The cultivation process took 12 h in order to obtain a liquid suspension culture with the cloudiness reaching up to 0.5 under the McFarland standard (approximately 1–2 × 10^8^ of colony forming units (CFU) per mL).

#### 4.4.3. Determination of the Antibacterial Activity

The antibacterial activity of the plant extracts was evaluated using a disk diffusion assay [61]. Inoculation onto the LB solid medium was done by means of sterile cotton swabs immersed in a suspension of test microorganisms. The inoculation process was carried out in three directions by strokes and turning the cup three times by 120°, so that once the inoculation was completed, a continuous and uniform lawn of the bacterial culture would be formed.

Various concentrations of the extract (6.25, 12.5, 25, and 50 mg mL^−1^) were applied onto sterile filter paper discs to obtain a final concentration of 0.125, 0.25, 0.5, and 1 mg of extract per disc, respectively. Then, the disks impregnated with the plant extract were placed onto the inoculated medium and pressed with forceps until the disk surface was completely in contact with the medium. By way of the positive control, a kanamycin antibiotic disk (25 μg per disk) was chosen. The disk impregnated with 20 μL of 10% DMSO was used by way of the negative control.

Petri dishes were incubated in an incubator at 37 °C for 12 h. After incubation, the antibacterial activity was determined by measuring the inhibition zone in millimeters around the disk (diameter). The test was repeated three times to ensure reliability.

### 4.5. Statistical Analysis

The obtained data were statistically processed employing the SigmaPlot 12.3 (Systat Software GmbH, Erkrath, Germany) and OriginPro 9 (OriginLab Corporation, Northampton, MA, USA) software. The tables and graphs show the average values with an indication of the standard deviation (*n* = 3). In order to identify the statistically significant differences between the experiment scenarios, the data underwent a two-way analysis of variance (ANOVA). Owing to the fact that the two-way analysis revealed an accurate correlation between the two factors, i.e., the plant part and its stage of development (Table A1), a one-way analysis was performed independently for each factor. Prior to application of the analysis of variance, the data were inspected for normality using the Shapiro–Wilks test, as well as a test for homogeneity. As the discrepancy validation criterion, the Tukey’s HSD test was chosen at a significance level of *p* < 0.05. Correlation analysis was performed using the Pearson criterion. In order to determine the correlation in the accumulation of individual groups of phenolic compounds, a multivariate analysis was performed, employing the principal component analysis. An assessment of the loadings’ significance in the PCA was performed using the bootstrap method (N = 999) according to Peres-Neto et al. [62]. The 95% bootstrapped confidence intervals for the eigenvalues were evaluated using Past v. 4.01.

## 5. Conclusions

The fact that the greatest medicinal value in heather lies in flavonoids, whose quantitative content reaches peak values in all plant organs during the floral budding stage, makes it possible to recommend heather gathering just before flowering. Nevertheless, the results presented in the current paper appear to be just the first phase of the studies aimed at monitoring changes in the content of certain groups of phenolic compounds in heather at different growth stages. In order to confirm the statement that antioxidant activity of heather extracts is determined by the presence of phenolic compounds, further and more detailed studies of the profile and amount of particular phenolic compounds at different growth stages need to be carried out. Meanwhile, the data obtained through the antibacterial analysis revealed that the floral budding stage turns out to be the only stage during which antibacterial activity was detected simultaneously in all studied plant organs with leaves and stems being active against all tested bacteria, whereas roots appeared to be active against *B. subtilis* and rhizomes against *E. coli* respectively. Therefore, the findings of the conducted study prove the prospects of using heather as a medicinal raw material as well as extracting valuable biologically active substances from it.

## Figures and Tables

**Table 1:** Content of the phenolic compounds in different parts of heather collected at different growth stages.

Growth Stage	Plant Parts	Phenolic Compounds, mg g^−1^					
		TPC ^1^	TTC	TFC	TAC	PAs	THA
Vegetative	Leaves	14.52 ± 1.85d^2^	17.69 ± 0.39b	172.7 ± 7.0b	0.96 ± 0.043a	5.80 ± 0.12c	4.54 ± 0.08b
	Stems	27.46 ± 0.80b	3.09 ± 0.30d	120.5 ± 2.8d	0.32 ± 0.007b	6.27 ± 0.20b	3.32 ± 0.09c
	Roots	31.66 ± 0.53a	18.16 ± 0.04a	239.6 ± 8.3a	0.26 ± 0.013bc	7.75 ± 0.25a	5.73 ± 0.10a
	Rhizomes	24.53 ± 0.61c	15.72 ± 0.33c	136.9 ± 2.3c	0.25 ± 0.008c	5.87 ± 0.26c	3.56 ± 0.09c
Floral budding	Leaves	28.15 ± 0.76a	14.94 ± 0.45c	305.6 ± 3.4a	0.92 ± 0.043a	8.82 ± 0.21bc	4.73 ± 0.13a
	Stems	20.47 ± 0.34d	12.39 ± 0.19d	112.6 ± 5.8d	0.30 ± 0.025c	6.20 ± 0.11d	3.17 ± 0.08c
	Roots	27.58 ± 0.34b	21.72 ± 0.86a	245.7 ± 8.3b	0.32 ± 0.015bc	9.21 ± 0.15ab	4.72 ± 0.04a
	Rhizomes	25.35 ± 1.10c	17.12 ± 0.30b	176.2 ± 4.1c	0.41 ± 0.006b	8.45 ± 0.20c	4.48 ± 0.13b
Flowering	Leaves	26.88 ± 0.11b	15.79 ± 0.11c	210.8 ± 2.2b	1.17 ± 0.017b	6.77 ± 0.21b	5.34 ± 0.05b
	Stems	24.06 ± 0.15d	14.95 ± 0.05d	93.5 ± 0.6d	0.81 ± 0.012c	4.55 ± 0.20c	3.11 ± 0.08d
	Roots	26.18 ± 0.32c	17.85 ± 0.41a	139.4 ± 2.2c	0.23 ± 0.002d	6.95 ± 0.30b	3.71 ± 0.10c
	Rhizomes	20.23 ± 0.04e	12.71 ± 0.09e	58.7 ± 1.1e	0.21 ± 0.005d	3.98 ± 0.21d	2.22 ± 0.08e
	Flowers	27.88 ± 0.18a	16.78 ± 0.12b	263.2 ± 1.1a	5.88 ± 0.026a	9.18 ± 0.11a	5.73 ± 0.12a
Seed ripening	Leaves	32.67 ± 0.12a	11.66 ± 0.14e	165.6 ± 3.5a	1.56 ± 0.017b	5.93 ± 0.26c	4.44 ± 0.08b
	Stems	26.58 ± 0.19e	11.95 ± 0.09d	65.8 ± 3.3e	0.42 ± 0.014c	5.02 ± 0.31d	2.97 ± 0.10e
	Roots	28.77 ± 0.16d	17.01 ± 0.09b	119.3 ± 3.5c	0.09 ± 0.003e	7.67 ± 0.21a	4.03 ± 0.07c
	Rhizomes	30.13 ± 0.12b	17.30 ± 0.05a	115.0 ± 1.6d	0.19 ± 0.007d	7.06 ± 0.13b	3.38 ± 0.09d
	Seeds	29.77 ± 0.15c	16.74 ± 0.11c	145.9 ± 2.8b	2.83 ± 0.021a	7.24 ± 0.22b	4.86 ± 0.07a

^1^ TPC—total phenolics content; TTC—total tannins content; TFC—total flavonoids content; TAC—total anthocyanins content; Pas—proanthocyanidins; THA—total hydroxycinnamic acid content. ^2^ Data were evaluated via one-way ANOVA separately for each growth stage followed by a Tukey test. Different letters indicate significant differences among the different heather parts at *p* ≤ 0.05.

**Table 2:** Correlation matrix with the Pearson coefficient values for the phenolic compounds and antioxidant activity of the heather extracts.

Parameters	TPC ^1^	TTC	TFC	TAC	PAs	THA	AOA (DPPH)	AOA (FRAP)
**TPC**	1	0.17	0.41 *	0.22	0.55 **	0.45 *	0.65 **	0.77 **
**TTC**		1	0.41 *	0.11	0.48 *	0.47 *	0.15	0.09
**TFC**			1	0.41 *	0.81 **	0.86 **	0.52 **	0.37 *
**TAC**				1	0.64 **	0.54 **	0.75 **	0.44 *
**PAs**					1	0.74 **	0.47 *	0.27
**THA**						1	0.68 **	0.40 *
**AOA (DPPH)**							1	0.63 **
**AOA (FRAP)**								1

^1^ TPC—total phenolics content; TTC—total tannins content; TFC—total flavonoids content; TAC—total anthocyanins content; PAs—proanthocyanidins; THA—total hydroxycinnamic acids content; AOA—antioxidant activity according to the DPPH (1,1-diphenyl-2-picrylhydrazyl) and FRAP (ferric reducing antioxidant power) assays. ** Correlation is significant at *p* ≤ 0.01; * correlation is significant at *p* ≤ 0.05.

**Table 3:** Antibacterial activity of heather extracts (the inhibition zones include the diameter of the disk—6 mm).

Growth Stage	Plant Parts	Inhibition Zone, mm							
		*E. coli*				*B. subtilis*			
		Extract Concentration, mg mL^−1^				Extract Concentration, mg mL^−1^			
		50	25	12.5	6.25	50	25	12.5	6.25
Vegetative	Leaves	9 (++)	8 (+)	7 (+)	–	–	–	–	–
	Stems	11 (+++)	8 (+)	8 (+)	7 (+)	7 (+)	7 (+)	6.5 (+)	–
	Roots	–	–	–	–	–	–	–	–
	Rhizomes	–	–	–	–	–	–	–	–
Floral budding	Leaves	9 (++)	8 (+)	8 (+)	7 (+)	10 (++)	9 (++)	8 (+)	–
	Stems	8 (+)	7.5 (+)	7 (+)	6.5 (+)	10 (++)	9 (++)	7 (+)	–
	Roots	–	–	–	–	9.5 (++)	8 (+)	7 (+)	–
	Rhizomes	9 (++)	8 (+)	7 (+)	6.5 (+)	–	–	–	–
Flowering	Leaves	–	–	–	–	–	–	–	–
	Stems	7 (+)	7 (+)	6.5 (+)	–	–	–	–	–
	Roots	–	–	–	–	9 (++)	8 (+)	7 (+)	6.5 (+)
	Rhizomes	–	–	–	–	–	–	–	–
	Flowers	7.5 (+)	7 (+)	7 (+)	6.5 (+)	–	–	–	–
Seed ripening	Leaves	9 (++)	8 (+)	7 (+)	7 (+)	–	–	–	–
	Stems	–	–	–	–	–	–	–	–
	Roots	12 (+++)	10 (+)	7.5 (+)	7 (+)	10 (++)	8 (+)	7.5 (+)	7 (+)
	Rhizomes	–	–	–	–	–	–	–	–
	Seeds	9 (++)	8 (+)	8 (+)	7 (+)	–	–	–	–

«+»—6–8 mm; «++»—9–10 mm; «+++»—11–12 mm; «–»—no inhibition zone was observed.

## Appendix A

**Table A1:** Results of the two-way ANOVA for phenolic compounds content and antioxidant activity of heather extracts.

Main Effects	Factors	TPC ^1^	TTC	TFC	TAC	PAs	THA	AOA (DPPH)	AOA (FRAP)
Plant part (P)	Leaves	249 b	15.0 c	214 a	1.16 a	6.8 b	4.8 a	275 a	227 a
	Stems	229 d	10.6 d	98 d	0.46 b	5.5 d	3.2 d	159 d	199 b
	Roots	265 a	18.7 a	186 b	0.23 d	7.9 a	4.6 b	219 b	188 b
	Rhizomes	240 c	15.7 b	122 c	0.26 c	6.3 c	3.4 c	171 c	213 a
Growth stage (G)	Vegetative	228 c	13.7 d	167 b	0.45 d	6.4 b	4.3 a	172 d	202 b
	Floral budding	250 b	16.5 a	210 a	0.49 c	8.2 a	4.3 a	193 c	206 b
	Flowering	231 c	15.3 b	126 c	0.61 a	5.6 c	3.6 c	214 b	181 c
	Seed ripening	274 a	14.5 c	116 d	0.56 b	6.4 b	3.7 b	246 a	239 a
Significance	P	*	*	*	*	*	*	*	*
	G	*	*	*	*	*	*	*	*
	P*G	*	*	*	*	*	*	*	*

^1^ TPC—total phenolics content; TTC—total tannins content; TFC—total flavonoids content; TAC—total anthocyanins content; PAs—proanthocyanidins; THA—total hydroxycinnamic acids content; AOA—antioxidant activity according to the DPPH (1,1-diphenyl-2-picrylhydrazyl) and FRAP (ferric reducing antioxidant power) assays. Different letters indicate significant difference among mean values according to a Tukey test at p ≤ 0.05. Asterisks indicate the significantly influential factors (*p* ≤ 0.001).
